# Beyond diversion: the economic and institutional impact of criminal mediation in Thailand

**DOI:** 10.3389/fsoc.2026.1857910

**Published:** 2026-09-08

**Authors:** Kiatanantha Lounkaew

**Affiliations:** Faculty of Economics, Thammasat University, Bangkok, Thailand

**Keywords:** access to justice, alternative dispute resolution, cost-effectiveness, criminal mediation, litigation economics, restorative justice, SDG 16, sustainable governance

## Abstract

**Introduction:**

Concerns over the workload of Thailand’s criminal justice institutions have returned mediation to policy debate, particularly for disputes in which the injury is modest but the administrative effort is not. This study asks what economic difference emerges when such cases are handled through mediation rather than through the usual criminal process.

**Methods:**

The analysis draws on two components of a single arrangement with Thailand’s Office of Justice Affairs: anonymized microdata from a field survey of 850 respondents across five provinces selected by stratified sampling on the Human Achievement Index, and administrative records of agency budgets, caseloads, and procedures. Private cost components are survey means and government components are budget-to-caseload ratios, all expressed in 2026 prices. Uncertainty is quantified by Monte Carlo simulation with 10,000 replications, and robustness is tested under four alternative assumptions about wages, travel, and legal representation.

**Results:**

A single minor case processed through the mainstream route costs approximately 3,828 U.S. dollars, whereas mediation resolves comparable matters for approximately 103 dollars, a reduction of about 97 percent that holds across four offense categories. The simulation yields a mean mainstream cost of 3,735 dollars with a 95 percent interval of [3,703, 3,811], a mean mediation cost of 59 dollars with an interval of [40, 122], and a mean reduction of 98.4 percent with an interval of [96.7, 98.9]. The reduction never falls below 94 percent under any sensitivity scenario.

**Discussion:**

Read within a litigation-decision framework, a difference of this magnitude would alter disputants’ incentives if they faced both options, because it widens the range within which settlement is mutually preferable to adjudication. The institutional implication carries comparable weight, since each case diverted from the adversarial track removes paperwork and scheduling pressure from police, prosecutors, and courts. Mediation on this evidence is not an auxiliary service but an instrument for keeping an overloaded system functional, an aim aligned with Sustainable Development Goal 16.

## 1 Introduction

With countries battling escalating caseloads and limited public budgets, questions regarding how justice systems manage routine criminal disputes have become more urgent. The question is not just whether institutions can process cases at all, but also whether they can operate without undermining fairness or draining the administrative capacity on which more serious matters rely. These divisions have been particularly prominent in many developing and middle-income countries, with institutional imperatives growing faster than the resources needed to meet them.

In this respect, Thailand’s experience is instructive. Its criminal justice system, which routes even the most straightforward offenses through police investigation, prosecutorial screening, and adjudication in the courts, currently processes a steady stream of low-level cases, which continually puts pressure on the police, prosecutors, and courts. For anyone who is caught up in this system, it is rarely simple to know what to do.

Victims often have to return several times to police stations for numerous rounds of questioning, queue in cramped court corridors for hours, pay for travel costs, and lose income for each visit. Offenders take the same route, often turning to attorneys to help them make their way through the procedural maze.

These exchanges generate accumulated costs: Police officers working on case files that last into the evening; prosecutors settling for unfinished investigations; and judges sitting on dockets packed with cases that are procedurally arduous yet routine. The net effect is that more elaborate and serious issues in life compete for space with those that could have relatively easy and less demanding solutions.

Reparative efforts and criminal mediation have found their way back into the spotlight in this context. As such, they provide a different route to resolve disputes, which, for the most part, only have a formal legal character when they arise through human interaction, friction between neighbors, casual property loss, or traffic troubles.

A long line of research has accumulated since Braithwaite’s initial work on reintegrative shaming to empirical work by Zehr (1990), Latimer et al. (2005), Bergseth and Bouffard (2007), and Bouffard et al. (2017). These authors argued that structured dialogue allows victims to express harm, encourages offenders to take responsibility, and rebuild relationships that were disrupted by a dispute. A similar trajectory is echoed internationally as well: both the United Nations Basic Principles and the Council of Europe’s Recommendation R(99)19 caLL for restorative approaches when participation is voluntary, and the nature of the harm is suitable for non-adversarial resolution (Council of Europe, 1999; United Nations, 2000; Wardianti et al., 2026; Zurnetti and Muliati, 2022).

Although existing scholarship tends to focus more often on the individual and psychosocial benefits of restorative practices, a parallel institutional argument is emerging. Mediation does not have to prepare multilayered case files as adversarial proceedings do, nor does it rely on multiple hearings that spread over months. Instead, disputes may be transferred to a single structured meeting presided over by trained mediators. These kinds of differences in processes are important in a system where caseloads are chronically increasing and where the ability to deal with minor issues at low administrative costs means that limited resources are allocated to crimes which require full adjudication.

This institutional logic aligns closely with the aims of the United Nations’ Sustainable Development Goal (SDG) 16, which links access to justice to the long-term health of public institutions and their governing capacity. Thailand already possesses a substantial mediation infrastructure: community justice centers handle local disputes, provincial justice offices maintain trained mediators, and courts operate annexed mediation programs for cases already within the judicial pipeline. Earlier assessments have suggested that mediation is significantly cheaper than conventional criminal processing. However, most of this evidence is now more than a decade old, predating the sharp escalation of institutional costs in Thailand, as well as the wider application of contemporary law and economics, access to justice, and sustainable governance frameworks to criminal justice systems.

This study addresses this gap by comprehensively reassessing the costs of processing minor criminal cases in Thailand. Using survey microdata and administrative data obtained from the Office of Justice Affairs (OJA), it recomputes the full cost of handling an average minor criminal case in monetary terms. The analysis incorporates travel expenses, opportunity costs of time, legal expenditures, and institutional labor and updates all values to 2026 using the consumer price index. The result provides the most recent comprehensive comparative estimate available of the cost of resolving minor cases through adversarial procedures versus mediation, with underlying quantities drawn from 2016 and 2017 and all monetary values expressed in 2026 prices.

The empirical findings are then interpreted through an economic model of litigation and settlement, enabling evaluation not only of cost differentials themselves but also of how those differences shape the incentives that condition parties’ behavior. This study makes three major contributions to the literature. First, it provides one of the most recent and detailed cost comparisons of formal criminal processing and mediation in Southeast Asia. Second, it links institutional cost structures to established theories of litigation, deepening understanding of how procedural design influences disputant behavior. Third, it situates Thailand’s experience within global debates on sustainable justice and institutional resilience, showing that mediation is not merely a mechanism for accelerating case resolution but a means of reallocating justice-sector investment in ways consistent with long-term institutional strength.

## 2 Literature review and conceptual framework

The topics discussed in this study are not tied to a particular disciplinary tradition. Instead, they find themselves situated between multiple studies that have developed in parallel, each with its own lexicon and analytic concerns. Restorative justice scholarship focuses on interpersonal dynamics and community processes; access-to-justice research has taken a more institutional and pragmatic stance; the economics of litigation has explored incentives and cost structures, while more recent scholarship on institutional sustainability considers the long-term capacity of the system.

Despite their differences, the research shares the following commonality at its center. The trajectory of a dispute through the justice system and the baggage along that trajectory determine who gets to operate within the system and how the system changes the way it works over time.

This section is not intended to be a full review of these bodies of work. Instead, the following subsections lay the analytical framework for the empirical analysis and demonstrate how a synthesis of insights from these studies can be linked toward mediation as both a procedural alternative and institutional design option.

### 2.1 Restorative justice and criminal mediation

As a response to the normal ways in which traditional justice characterized harm, restorative justice arose out of critique. Rather than viewing crime as essentially an infringement on authority and order, early criminologists Braithwaite (1989) and Zehr (1990) contended that crime ought to be situated in living, relationships, and communities experiencing harm. One implication of this reconceptualization is that we shifted our attention beyond mere punishment and toward processes that enable victims to be confronted face-to-face with perpetrators using a trained and empathetic facilitator able to navigate between both emotional complexity and factual ambiguity.

Over time, extensive empirical studies have demonstrated that the outcomes produced by restorative processes, when designed and executed carefully, diverge dramatically from those produced by adversarial processes. Previous studies on victim satisfaction and perceptions of procedural fairness, such as Bergseth and Bouffard (2007), reported more positive perceptions. Recent experimental and quasi-experimental studies support these findings. For instance, a Danish trial by Van Mastrigt et al. (2024) showed through a rigorous research design that structured restorative experiences can enhance participants’ experiences without compromising the legal legitimacy of treatment (see also Andrews and Eide, 2026; Wood and Suzuki, 2024).

In scholarship, restorative justice has recently been reframed as institutional, rather than merely interpersonal. Such scholars as Procter-Legg et al. (2024) have focused less on relational repair on their own and instead on the ways in which restorative programs redistribute authority and change procedural trajectories in justice systems (see also Marder, 2022; Nogalska et al., 2026).

However, the institutional turn draws cautionary lines. As Braithwaite (2022) observed, restorative practices must not be marginal add-ons to adversarial systems or arbitrary extras only; rather, regulatory design must be made permanent by integrating them into regulatory frameworks at all levels of decision-making in the architecture of a durable restorative architecture. Similarly, Daly (2002) cautioned that restorative efforts run the risk of reproducing existing power imbalances instead of reducing them without institutional rooting (see also Yang et al., 2026).

This tension is evident in Thailand. Community justice centers and provincial mediation units work and provide assistance, but they work primarily alongside and not as part of the core criminal justice apparatus. Restorative practices have such a function but remain limited to the long-term nature of the system. Collectively, the literature offers both a moral justification for mediation and an institutional justification for treating it as a more expansive incentive environment, rather than just an alternative procedural tool.

### 2.2 Access to justice, institutional design, and sustainable governance

Literature on access to justice has further expanded the analytical framework discussed in the previous subsection. This research does not simply ask whether individuals have formal legal rights, but whether the institutional machinery by which this right is realized in practice can be managed. As Taylor Poppe (2021) suggested, the complexity of the procedure is already sufficient to act as a barrier: queues can stretch, hearings can become bloated, procedures are unclear, and outcomes are difficult to predict for the recipient. Such friction not only inconveniences users but also systematically discourages engagement with formal legal routes.

Barendrecht et al. (2006) conceptualized justice services as bundles of attributes, including price, time, availability, and predictability, whose configuration determines whether ordinary users will be able to effectively use them (see also Illankoon et al., 2022; Gromova and Ferreira, 2025). From this standpoint, access to justice is not necessarily a binary condition but rather a result of institutional structuring decisions. When costs, delays, or uncertainty are intolerable, formal justice is legal but rarely achievable for the vast majority.

When these micro-level frictions are considered together, a sustainability dimension arises. SDG 16 encompasses the tenet that fairness, integrity, and access can only be sustained if institutions can handle their caseloads without overwhelming their personnel or resources. To this extent, a special difficulty involves smaller criminal cases that require multiple procedural steps with little harm.

Using cross-country panel data, Deseau et al. (2025) demonstrated that jurisdictions associated with high procedural burdens or restricted access domains tend to record weakened institutional performance and inefficient resource allocation over time (see also Isiaka et al., 2025). According to this perspective, procedural accessibility is not on the fringes but at the center of governance itself.

Viewed through this lens, mediation is not merely an exercise for resolving disputes. Disputants abbreviate process paths. Rather than engaging in successive investigative and judicial stages, most encounters are scheduled for a single facilitation-managed meeting. Travel, waiting time, and opportunity costs decreased accordingly. These savings compound at the institutional level, as well as fewer investigative hours, fewer prosecutors’ files, and less heat on court dockets. Mediation thus sits squarely within the logic of sustainable governance, upholding access, and fairness while reducing the cumulative loads experienced by justice institutions over time.

### 2.3 Law-and-economics models of litigation and settlement

The economics of litigation provides a formal foundation for analyzing how cost structures influence dispute resolution. The canonical Landes model (Landes, 1971) conceptualizes the plaintiff’s expected payoff from trial as.

$${\pi_{P}}^{\text{trial}}=p_{P}J-C_{P}$$

$${\pi_{D}}^{\text{trial}}=-p_{D}J-C_{D}$$

$$S_{\min}=C_{P}-p_{P}J$$

$$S_{\max}=p_{D}J-C_{D}$$

This framework highlights that litigation costs shrink or expand settlement regions. High costs narrow the region and increase the likelihood of a trial, whereas low costs expand it and increase the likelihood of negotiated agreements. Gelbach (2018) showed that procedural rules influence the types of cases that enter litigation by altering the expected costs, while Kobayashi (2014) emphasized that cost structures shape not only settlement probabilities but also dispute trajectories across forums (see also Tiwari and Totuka, 2026).

To illustrate this, consider a stylized dispute with an expected judgment value J = 1,000. If the plaintiff estimates a 50 percent chance of success and faces litigation costs of Cₚ = 600, then:

$$S_{\min}=600–0.5\times 1,000=100$$

$$S_{\max}=0.4\times 1,000–700=-300$$

Under mediation, where costs fall to approximately C = 50:

$$S_{\min}=50–0.5\times 1,000=-450$$

$$S_{\max}=0.4\times 1,000–50=350$$

### 2.4 Conceptual extension: mediation as a structural alternative

In Thailand, disputants choosing how to resolve minor criminal disputes face a bifurcated institutional landscape. One pathway is the mainstream criminal process, where the costs of Cₚᵐᵃᵉⁿ and Cᵈᵐᵃᵉⁿ reflect travel demands, waiting times, legal representation, administrative requirements, and sustained interactions with the police, prosecutors, and courts. The alternative is mediation, in which the costs Cₚᵐᵉᵈ and Cᵈᵐᵉᵈ are shaped by a single facilitated encounter and minimal administrative overhead. The empirical analysis in Section 5 suggests the following:

$$C_{\mathrm{med}}\ll C_{\text{main}}$$

**Figure 1:**
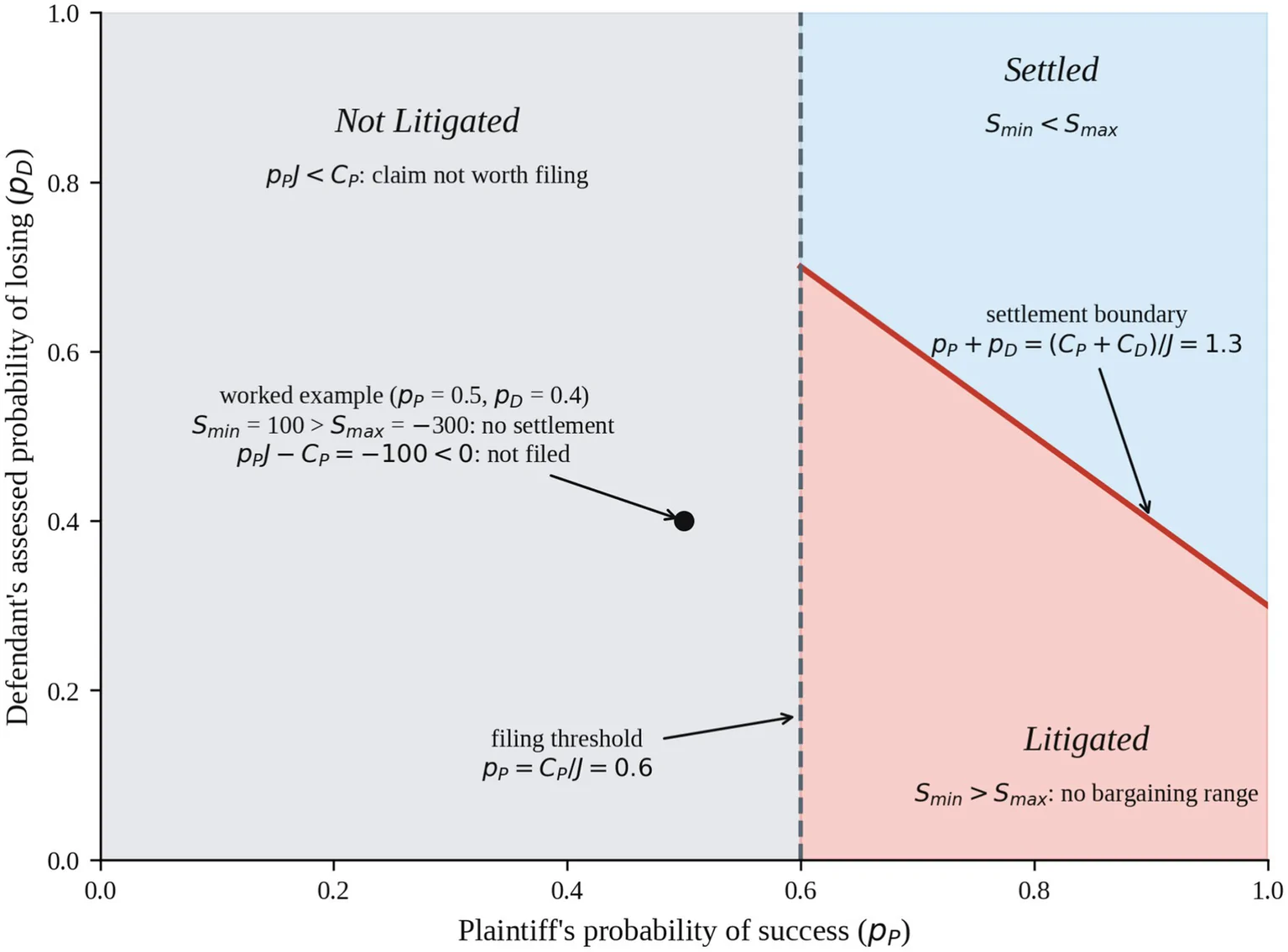
Litigation decision under mainstream criminal procedure.

**Figure 2:**
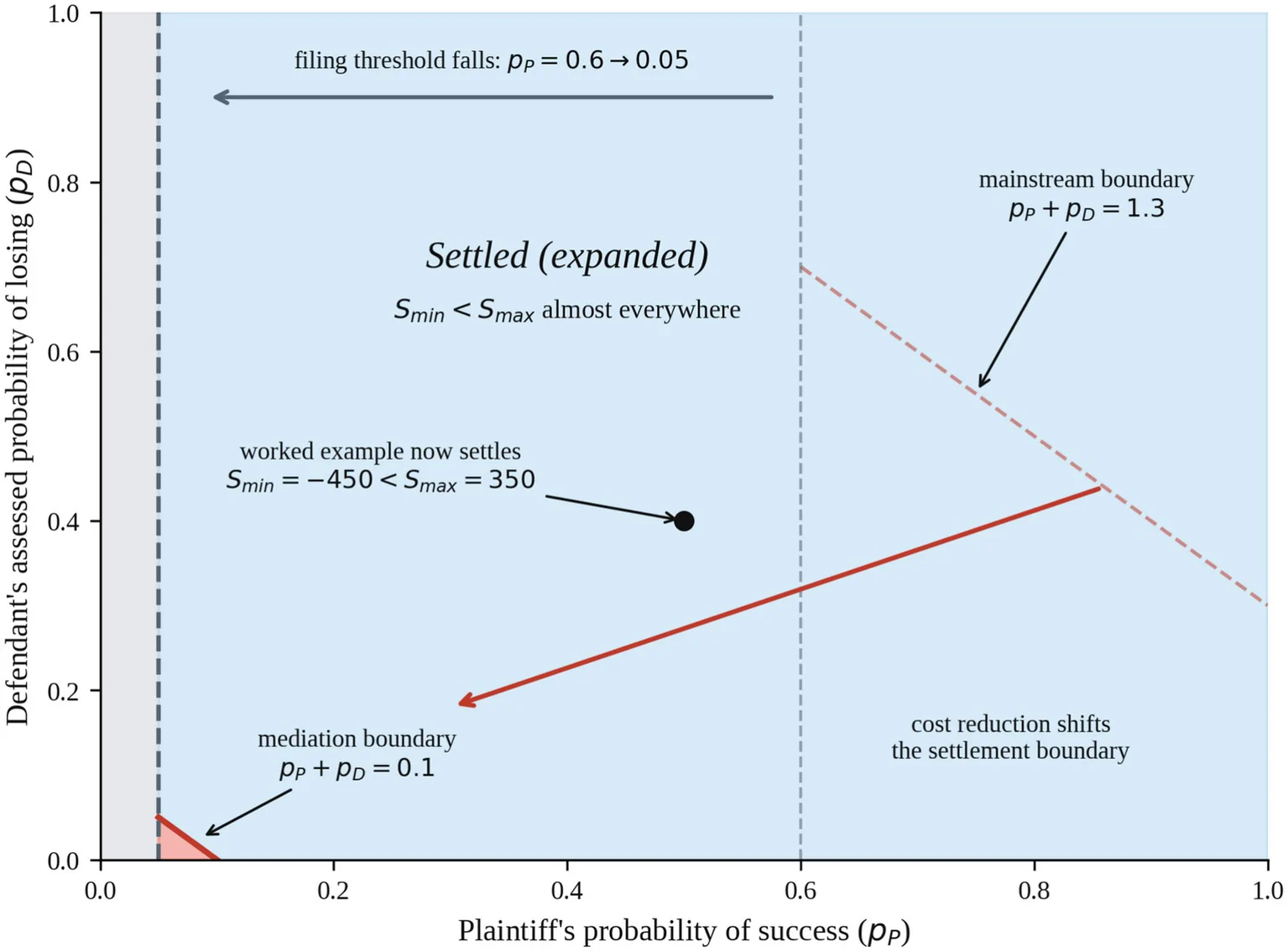
Effect of cost reduction on litigation decision.

This conceptual extension is visually represented in two analytical figures. Figure 1 depicts the high-cost adversarial decision space where the settlement region is narrow or non-existent. Figure 2 illustrates how mediation widens the settlement region by compressing both sides’ cost curves. Although presented schematically, these figures mirror the empirical reality documented in Section 5. They provide a theoretical rationale for why mediation substantially alters dispute trajectories.

The decision space is defined by the plaintiff’s probability of success (horizontal axis) and the defendant’s assessed probability of losing (vertical axis), following the model in Section 2.3 with *J* = 1,000, *C_P_* = 600, and *C_D_* = 700. Gray marks cases not litigated because the expected judgment does not cover the plaintiff’s costs (*p_P_* < *C_P_*/*J* = 0.6). Red marks the litigated zone, where no bargaining range exists (*S_min_* > *S_max_*). Blue marks the narrow settlement zone above the boundary *p_P_* + *p_D_* = (*C_P_* + *C_D_*)/*J* = 1.3. The dot marks the worked example from Section 2.3, which under mainstream costs is not pursued.

Under mediation costs (*C* = 50 per party), the settlement boundary shifts from *p_P_* + *p_D_* = 1.3 (dashed) to *p_P_* + *p_D_* = 0.1, and the filing threshold falls from *p_P_* = 0.6 to *p_P_* = 0.05. The blue settlement zone expands to cover nearly the entire decision space, while the red litigated zone and the gray not-litigated strip shrink to slivers. The worked example from Section 2.3, unresolvable under mainstream costs, now falls well inside the settlement zone.

### 2.5 Sustainable justice, institutional resilience, and SDG 16

Debates on justice reform have increasingly embraced a systems approach, interrogating how institutions hold up over long stretches of time instead of how they perform in individual cases. The phrase ‘sustainable justice’ emerged from these changes. It is less of a theory than a concern shared across various lines of research: A justice system must continue to be practical and credible as caseloads increase, budgets fluctuate, and public expectations move in different directions.

SDG 16 neatly addresses this concern, if indirectly, by placing effective and accountable institutions on the same footing as peace and access to justice. The literature on institutional performance increasingly connects access to justice with larger indicators of governance quality and long-term system resilience (Haggard and Tiede, 2014; OECD, 2025; Taggart, 2022). These findings confirm that sustainable justice institutions balance fairness, accessibility, and operational capacity over a long period.

Mediation addresses this conversation in a surprisingly pragmatic way. It shortens the procedural process, sometimes to one meeting, breaking the build-up of administrative work, which is usually accompanied by even minor criminal charges. A mediated case does not create a cycle of file transfers, follow-up investigations, or delayed hearings within adversarial systems. Each resolved case matters independently. However, the aggregate effect is not trivial.

When a steady flow of small disputes is mediated instead of processing through a formal pipeline, investigators have time to breathe, prosecutors have fewer mundane files to deal with, and courts have lighter dockets. It is this silent shift in administrative burden, not a massive transformation, which makes mediation a means of promoting system resilience.

Sustainable justice is a matter of institutional stamina as a right or procedure. A system that burns itself dry over small matters will eventually underperform in areas of adjudication that require adjudication to be effective. Mediation protects against drift by reducing long-run operational strain. The analyses in Section 5 are read with this background in mind because the documented cost differences ultimately feed into questions of institutional viability rather than cost alone.

## 3 Thai criminal justice context

Thailand’s criminal justice system is an assemblage of institutions, each with statutory duties and administrative habits. This appears together in formal diagrams; however, in practice, the experience of taking a case through this form appears fragmented and, at times, slow. Each aspect of the process is handled by the police, prosecutors, judges, and post-trial agencies, and matters can ignite a series of interactions that seem disproportionate to the underlying problem. Given the seriousness of minor crimes at the heart of this investigation, the system architecture yields a rigorous process that is not easily rationalized in terms of its efficiency. We hope that any effort or analysis to explain the cost comparisons offered later will start with an accurate description of what these institutions do in effect.

A complaint generally begins at a police station, and the initial process is far larger than most people envision. Officers must record the allegations in detail, draw statements, and determine what provisions of the Criminal Code may apply. Even in trivial matters, whether a shove that turned into an altercation, a quarrel between neighbors, or a cracked cell phone screen, the officer is expected to prepare an investigation file. It can contain interviews, witness notes, and paperwork that, although standard, takes time to complete. Victims and suspects must appear in person, often in multiple appointments, and travel and waiting require their own resources. Even a short custodial period for a suspect creates the need for judicial oversight and another procedural layer before the case may reach a certain distance.

After the police consider the file to be complete, they enter the Office of the Attorney General. Prosecutors examine the evidence, occasionally return the file for further work, and call the parties for clarification or exploratory settlement discussions. As significant as this stage is for filtering out weak or false complaints, it also prolongs the life of minor cases in ways that cause real costs to those who deal with them. For people who hold daily wage jobs or who live outside the province, each visit represents lost income and extra expenses.

If the prosecutor decides to proceed, the case winds up in a Court of First Instance. Court rhythms are dictated by packed calendars, and hearings for fewer cases can take place weeks or months apart. For victims and defendants alike, on a hearing day, it is routine for clerks and judges to find hours waiting to comb through an eternally shifting docket. Even when the ultimate sanction is small, it is difficult to achieve it on a light track. Minor cases involve the same machinery as serious cases, dragging officers, prosecutors, and court personnel into work, which is often out of scale with the harm involved.

Over time, especially as the number increases, this steady pull manifests itself in slower calendars, backlogs, and the familiar feeling that the system is perpetually over capacity. These are not abstract design flaws; rather, they determine the decisions that people make within a system. Victims who depend on wages every day or who live beyond the district center may find each necessary appearance chip away from their ability to get and stay involved. The defendants put their bodies under a different type of pressure, one based on the uncertainty and repetition of the process, instead of the penalty that looms at the end.

For institutions, the difficulty is in dividing the little administrative time allotted for matters that truly require full attention or those that just happen to cross into the formal process. Ironically, however, a small number of cases can be disproportionate in terms of the time taken to arrive. Such structures are not just in the background but exert a large influence on the way people behave and how institutions function. For victims with restricted time or transportation, the cost of remaining in the system may be unaffordable. Defendants may also feel the pressure of more than one time and/or repeated appearances and the potential for longer periods of uncertainty than punishment can be overwhelming. Organizations also have to spend too much administrative time on a combination of serious and trivial problems. Tangkitvanich et al. (2011) and Sindecharak and Kittiratanapan (2020) observed these inefficiencies for over a decade (see also Pomerantz and Altgilbers Roweton, 2024). In response, Thailand has developed multiple tiers of mediation.

The most local tier is the center for community justice. These centers are based on district-level agencies or local administrations and employ a combination of volunteers and justice officials. Their mission is to resolve disputes rooted in everyday life: vendors’ disagreements, loud arguments between neighbors, and small drinking assaults. Because mediators are aware of the local context or learn it quickly, these encounters are more personal and often more productive.

The second tier, the provincial justice office, is more formal. These offices have trained mediators to understand negotiations, legal processes, and structured dialogue. They receive referrals from the police and prosecutors, and walk-in requests from people who want to avoid the adversarial route. Sessions are often more formal than those at community centers, and agreements are formally documented. Prosecutors frequently weigh these records to determine whether they need to take formal action.

The third tier involves court-annexed mediation. Here, the case settled into the judicial pipeline once, and the stakes are different. Judges oversee the process at a distance; lawyers are more likely to be there, and its tenor is shaped by the knowledge that the trial is still an immediate alternative if talks fail. Even so, the aim is the same across tiers: to reduce conflict, promote restitution or an apology where justified, and ease the burden on courts.

When combined, they constitute an ecology parallel to the adversarial process. This difference is not only in the restorative ethos but also in its very different cost structures. Mediation collapses multiple visits into one, reduces the travel and waiting involved, and saves police and prosecutors the detailed preparation of files required by the conventional system.

With respect to the litigation-decision model described above, this effect means that the cost functions Cₚᵐᵉᵈ and Cᵈᵐᵉᵈ fall accordingly, changing the likely options for victims and defendants such that they all go in the direction of settlement. These pathways matter from a sustainability standpoint. Diversion reduces duplication; avoids trivial matters that consume disproportionate hours inside the institution; and frees the police, prosecutors, and courts to deal with cases where adjudication is necessary.

For a body that must keep up with severe caseloads every year, even small shifts in how work is divided can make a difference. They add up quietly and, over time, contribute to whether the system remains responsive or becomes routinely congested. Keeping these pressures in view is critical for interpreting the cost patterns later in Section 5, as well as for trying to understand the reasons why some policy choices appear to carry greater weight than they did initially.

## 4 Materials and methods

The empirical work in this study was based on material obtained from the OJA under the Ministry of Justice. The OJA provided two complementary components under a single restricted arrangement: anonymized case-level microdata from a field survey of 850 respondents conducted in 2017 as part of the OJA-commissioned study, and administrative material comprising internal procedural notes, budget and caseload statistics, and operational descriptions of how criminal cases normally move through Thai institutions. Taken together, these sources allowed reconstruction of the ordinary steps involved in processing a minor case and, by extension, the private and institutional costs attached to each step. The aim of this section is to explain what the data contain, how the relevant concepts were defined, and how the cost estimates used in the study were produced. Because the findings directly inform policy, the discussion highlights the assumptions that shape the modeling exercise.

### 4.1 Sample description

The field survey covered 850 respondents who had direct experience with the criminal justice system, sampled from five provinces selected by stratified sampling on the UNDP Human Achievement Index (HAI) to ensure coverage across development tiers. The provinces and their shares of the sample were Chiang Mai (25 percent, n = 213, high HAI), Surat Thani (22 percent, *n* = 187, medium HAI), Ubon Ratchathani (20 percent, *n* = 170, low HAI), Chonburi (18 percent, *n* = 153, high HAI), and Phitsanulok (15 percent, *n* = 128, low-medium HAI). The sample size was determined using the Rea and Parker (1997) formula at 99 percent confidence with a 4.41 percent margin of error.

Of the 850 respondents, 622 (73 percent) had been processed through the mainstream criminal pathway and 228 (27 percent) through mediation. Cases were distributed across four offense categories: minor bodily harm (35 percent, *n* = 298), property damage and minor theft (25 percent, *n* = 212), traffic disputes (22 percent, *n* = 187), and interpersonal disputes including neighbor and workplace conflicts (18 percent, *n* = 153). This distribution reflects the proportional allocation of cases across provinces and offense types in the underlying administrative records. Table 1 summarizes the sample by province, pathway, and offense category.

**Table 1:** Sample description: province, pathway, and offense category (*N* = 850).

Characteristic	*n*	%	
Panel A: province (by HAI tier)			
Chiang Mai (high)	213	25.1	
Surat Thani (medium)	187	22.0	
Ubon Ratchathani (low)	170	20.0	
Chonburi (high)	153	18.0	
Phitsanulok (low-medium)	128	15.1	
Panel B: pathway			
Mainstream criminal	622	73.2	
Mediation	228	26.8	
Panel C: offense category			
Minor bodily harm	298	35.1	
Property damage/petty theft	212	24.9	
Traffic disputes	187	22.0	

Cases were not randomly assigned to mainstream processing or mediation. Eligibility for mediation depends on offense type, the willingness of both parties, and the discretion of the referring authority. The cost comparison that follows is therefore a descriptive comparison of two procedural architectures rather than a causal estimate of the effect of pathway assignment. What the analysis identifies is the cost a representative case incurs under each architecture, not the effect of being assigned to one pathway rather than the other.

A large part of the empirical data comes from administrative material that maps the procedural logic of the criminal process. For each stage (police, prosecutors, and the Court of First Instance), the documents describe the routine tasks performed by officials, the typical number of appearances by complainants and suspects, and the hours ordinarily spent on investigation, review, and hearing preparation. These are real accounts; they reflect the duties prescribed in Thai criminal procedure and the workflow used in practice. Because nearly every procedural step consumes time or administrative labor, these descriptions serve as scaffolding through which institutional effort is translated into monetary value.

The survey microdata described in Section 4.1 complement the procedural material. These records, drawn from respondents whose mediation and mainstream cases were handled across the five study provinces, contain the elements required to compute the direct burdens on victims and offenders. They show how many trips a person makes, how far they travel for each visit, how long they wait on a typical day at a station or court, and the incidental expenses that arise along the way. The cases were coded by offense type, which allowed comparisons across four categories commonly deemed suitable for mediation: minor assaults, property damage or petty theft, traffic-related disputes, and interpersonal conflict. This granularity matters, because the personal and institutional burdens attached to each category differ.

Costs were broadly defined in this study such that the estimates capture the full economic weight of participating in the justice process. For private parties, the model includes travel expenses, the value of time spent waiting and traveling, income lost from missed work, and fees associated with documents or legal representation. The time component is valued using a shadow price anchored to the prevailing Thai wage conditions and updated to 2026 values using consumer price inflation. Formal legal fees are included only in situations in which adversarial proceedings commonly require representation, which, in Thai practice, extends even to minor matters.

Institutional costs were derived by converting staff time into monetary value. For police, this includes the hours spent recording complaints, interviewing victims and suspects, reviewing evidence, and communicating with prosecutors. For prosecutors, the cost components include file reviews, supplementary evidence requests, and preparation required for hearings. The courts incur scheduling, file management, and procedural oversight costs. The OJA provided administrative estimates for each of these components, which were then cross-checked against published assessments of institutional workload.

### 4.2 Cost construction and descriptive statistics

Two categories of cost data enter the analysis. Private cost components, including travel, accommodation, food, and communication expenses, were computed as arithmetic means from the field survey. The survey data exhibit substantial dispersion: for example, travel costs for visits to the police ranged from approximately 0.13 to 165 U.S. dollars in 2026 prices (4 to 5,000 baht) with a mean of approximately 6 U.S. dollars (188 baht) and a standard deviation of approximately 12 U.S. dollars (373 baht); travel costs for court appearances ranged from approximately 0.13 to 331 U.S. dollars (4 to 10,000 baht) with a mean of approximately 8 U.S. dollars (247 baht) and a standard deviation of approximately 19 U.S. dollars (565 baht).

Government cost components, by contrast, are deterministic ratios of annual agency budgets to annual caseloads. The police cost per case equals the 14,851.28 million baht justice-administration budget divided by 575,685 cases (491,140 criminal plus 84,545 traffic), yielding approximately 854 U.S. dollars in 2026 prices (25,798 baht) per case. The prosecutor cost per case equals 7,936.62 million baht divided by 2,190,000 cases, yielding approximately 364 U.S. dollars (10,986 baht) per case. The court cost per case equals 9,660 million baht divided by 1,826,160 cases, yielding approximately 175 U.S. dollars (5,290 baht) per case. Because these are population-level accounting identities rather than sample statistics, they carry no sampling variance by construction. This feature explains why the reduction percentages across offense types in Table 2 are so similar (97.0 to 97.3 percent): the government costs that dominate the total are invariant across categories, and the small differences reflect only the modest variation in private cost components.

**Table 2:** Disaggregated systemwide costs by mediation-eligible case type (U.S. dollars, 2026).

Case type	Mainstream cost	Mediation cost	Reduction (percent)
Minor bodily harm	4,012.00	118.50	97.0
Property damage and minor theft	3,765.40	110.40	97.1
Traffic disputes	3,544.90	96.70	97.3
Interpersonal disputes (e.g., neighbor, workplace)	3,671.50	101.20	97.2

Because the source material spans several years, all monetary figures were expressed in 2026 prices. The adjustments followed the standard CPI update.

$$\text{Cost}2026={\text{Cost}}_{\text{base}}\times (\mathrm{CPI}2026/{\mathrm{CPI}}_{\text{base}})$$

After inflation adjustment, all values were converted to U.S. dollars using the average exchange rate between January and July 2026, a period selected to smooth out short-term volatility and align with the conditions prevailing during the analysis.

The private cost data were collected through field surveys conducted in 2017 (B.E. 2,560), while the government budget figures used to compute institutional costs per case refer to fiscal year 2016 (B.E. 2,559). The shadow wage used for opportunity cost valuation is drawn from the National Statistical Office’s average monthly earnings of employed persons in 2016, equal to approximately 605 U.S. dollars per month in 2026 prices (18,286 baht) or approximately 27.50 U.S. dollars per day (831 baht) at 22 working days per month. This figure was updated to 2026 prices using the CPI ratio described above.

Case selection adhered to the legal definitions governing mediation eligibility. Only offenses involving minor injuries, small-value property loss, non-felonious traffic disputes, and relational or neighborhood conflicts were included. Cases involving weapons, narcotics, serious bodily harm, or threats to public safety were excluded, as they fell outside the legally defined mediation space. This ensured that the comparisons reflected the real choices available to disputants in the Thai legal system.

This empirical strategy involved several steps. First, the model constructed the full cost of mainstream processing for each of the four offense types by combining travel expenses, waiting-time valuations, legal fees, and institutional labor. Second, an analogous reconstruction was performed for mediation, using microdata on travel, session duration, and administrative involvement. Because mediation compresses the procedural path into a single event, these costs are markedly lower. Third, the two sets of figures were compared, producing percentage reductions that illustrate the scale of divergence between pathways.

Next, counterfactual diversion scenarios were simulated. These estimates indicate what would happen if 25, 50, or 75 percent of eligible cases were diverted to mediation in a notional cohort of 10,000 disputes. The simulations quantified the potential savings for both individuals and the state, and revealed how shifting caseloads altered institutional demand. Sensitivity tests varied the wage rates, travel distances, and legal fees to assess whether cost differences hold under alternative conditions.

Finally, the numerical results were interpreted using the conceptual model outlined in Section 2. The differences between Cₚᵐᵃᵉⁿ, Cᵈᵐᵃᵉⁿ, Cₚᵐᵉᵈ, and Cᵈᵐᵉᵈ were mapped onto the litigation-decision framework, highlighting how reductions in private and institutional costs expanded the region in which settlement became the rational choice. This integration forms a bridge between the empirical results in the following section and broader institutional arguments explored in Section 6.

## 5 Results

The numbers that emerge from the empirical work point in a single direction: the mainstream criminal process is far more expensive, in every sense, than mediation, and this pattern appears across all offense types examined. The size of the gap is striking, but what matters for present purposes is that it is also steady, appearing each time the calculation is reconstructed from a different perspective. The analysis reported in this section follows the structure described in the previous sections. It begins with the overall cost picture; unpacks the burdens on victims, offenders, and justice institutions; and finally turns to diversion and sensitivity exercises that help clarify the institutional consequences of shifting cases out of the adversarial pipeline.

To establish a baseline, this study first reconstructed the cost of handling a routine mediation-eligible case through conventional institutions. This reconstruction pulls together the strands described earlier: hours spent by police investigating and drafting files, the time prosecutors devoted to reviewing and refining those files, appearances before the Court of First Instance, the long waiting periods that accompany them, the travel and income losses absorbed by the parties, and the administrative work embedded throughout. After adjusting all components to 2026 prices and converting them into U.S. dollars, the cost of such a case was approximately 3,828 U.S. dollars. This figure is noticeably higher than the levels reported in earlier Thai studies, not because the earlier work was flawed but because costs have risen, and the present analysis counts a wider set of burdens that were previously left at the margins.

Mediation produces a different picture. Using the OJA survey microdata, which track travel, waiting, session time, and the limited administrative support involved, a standard mediation case costs approximately 103 U.S. dollars. When set alongside the mainstream estimate, this implied a reduction of approximately 97 percent. The difference does not hinge on one offense type or procedural quirk; it appears consistently across the full set of categories examined. The subsections that follow, together with the accompanying tables, provide the detailed results.

### 5.1 Costs of the mainstream criminal justice process

The mainstream process is characterized by multiple procedural layers that require repeated interactions with the police, prosecutors, courts, and occasionally legal counsel. These interactions generate direct and indirect costs. Table 3 summarizes the updated cost estimates for a typical mainstream case in U.S. dollars.

**Table 3:** Updated costs of the mainstream criminal justice process (U.S. dollars, 2026).

Procedural stage	Offender cost	Victim cost	Government cost
Police investigation and evidence review	40.91	—	773.93
Prosecutorial examination	23.65	—	329.58
Court of First Instance (trial hearings)	14.55	14.55	158.69
Court-annexed administrative day	7.26	7.26	104.13
Lawyer fees	900.00	900.00	—
Opportunity cost of lost income	274.23	274.23	—
Miscellaneous fees	3.00	3.00	—
Total private burden	1,263.60	1,198.04	—
Government total	—	—	1,366.33
Systemwide cost per case	3,827.97		

The weight of legal fees stands out most in the private cost figures. Even in cases that look minor on paper, defendants, and not infrequently victims, tend to hire lawyers simply to make sense of the process and guard against the risk of a case drifting to trial. This tendency rapidly pushes the cost curve upwards. The lost time is added to another layer. Many parties earn daily wages or live far from the courthouse; therefore, each appearance indicates income foregone or several hours spent on the road. On the institutional side, the burden is uneven. The police carry much of it. The investigation stage, with its interviews, paperwork, and coordination, absorbs more than half of the state’s expenditure on a typical minor case. Prosecutors closely follow with their own file reviews and clarifications. Courts shoulder a smaller share; however, because they handle large volumes of cases each year, the aggregate impact is small.

The lawyer fee used in this analysis, approximately 990 U.S. dollars per party in 2026 prices (30,000 baht), was elicited from focus group consultations with representatives of justice agencies in Bangkok and in each of the five study provinces. The estimate is consistent with published data on legal fees in the Thai system. According to the Justice Fund Committee Rules, enlisted lawyer compensation for criminal cases at the Court of First Instance ranges from approximately 165 to 1,655 U.S. dollars per case (5,000 to 50,000 baht). Lounkaew and Rieoraengkuson (2022), analyzing 1,546 cases handled by the Thai Justice Fund between 2012 and 2019, report a mean fee of approximately 712 U.S. dollars (21,512 baht) and a median of approximately 662 U.S. dollars (20,000 baht) for criminal cases; because these are subsidized legal-aid rates, market rates for privately retained counsel would be higher, placing the estimate used here within a plausible range.

### 5.2 Costs of provincial and community mediation

Mediation fundamentally alters this architecture. Sessions typically require a single visit and involve no formal legal representation unless parties independently choose to retain counseling and operate with minimal bureaucratic overhead. Table 4 summarizes the cost structure of a typical mediation case.

**Table 4:** Updated costs of provincial and community mediation (U.S. Dollars, 2026).

Cost component	Offender Cost	Victim Cost	Government Cost
Travel and time to mediation venue	9.00	9.00	21.90
Mediation session administration	—	—	12.00
Opportunity cost of lost income	24.00	24.00	—
Other minor expenses	1.50	1.50	—
Total private burden	34.50	34.50	—
Government total	—	—	33.90
Systemwide cost per case	102.90		

The overall systemic mediation cost was approximately 103 U.S. dollars. The absence of lawyer fees, near-elimination of travel to multiple institutions, and consolidation of procedural interactions into one facilitated encounter accounted for the majority of savings. The contrast between mainstream and mediation costs reflects not only streamlined procedures, but also the reallocation of institutional responsibility. Mediation shifts the locus of problem solving away from adversarial structures, engaging professional and volunteer mediators rather than the police, prosecutors, and courts.

### 5.3 Disaggregated costs by mediation-eligible case type

Although the preceding tables present modal estimates, the overall cost differential remains consistent across the different categories of minor criminal disputes. Table 2 summarizes the costs for the four major mediation-eligible case types.

Across the different offense types, the numbers settle into a pattern: mediation trims system-wide costs by well over 90 percent, and in most cases, by something close to ninety-seven. The exact figures move slightly from category to category; some disputes involve longer travel while others require more discussion around restitution, but none of these variations change the basic picture. Mediation is cheaper by a wide margin and not because of the special features of a particular case type. This result agrees with an earlier conceptual argument. When the underlying cost structure shifts dramatically, the space in which settlement becomes the sensible option widens, and people respond accordingly.

### 5.4 Counterfactual diversion scenarios

To understand the system-wide implications, Table 5 simulates the effects of diverting different proportions of mediation-eligible cases away from mainstream pathways. Using a cohort of ten thousand cases, the table models the total costs under 25, 50, and 75 percent diversion scenarios.

**Table 5:** Counterfactual diversion scenarios: systemwide savings (U.S. dollars, 2026).

Diversion rate	Cost under mainstream	Cost under mediation	Total savings
25 percent	9,569,925	257,250	9,312,675
50 percent	19,139,850	514,500	18,625,350
75 percent	28,709,775	771,750	27,938,025

Each row of Table 5 reports only the cost of the diverted cases, valued first under mainstream processing and then under mediation. The difference represents the saving attributable to diversion. The cost of the non-diverted remainder is unchanged and is not shown, because the purpose is to isolate the marginal saving from each additional tranche of diverted cases. The diversion costs are computed using offense-category-specific costs from Table 2, weighted by the sample proportions reported in Section 4.1, rather than the pooled average.

Under the modest diversion scenario, the system saves more than 9 million U.S. dollars. At 50 percent diversion, which is an attainable threshold given the existing infrastructure, savings exceed 18 million U.S. dollars. A 75 percent diversion threshold yields almost 28 million U.S. dollars in savings. These figures highlight the structural inefficiencies of the mainstream process and the potential of mediation to relieve institutional congestion, reduce fiscal pressure, and enhance the sustainability of justice institutions.

### 5.5 Sensitivity analysis

Table 6 presents the recalculated systemwide costs under four alternative assumptions. Under Scenario A, halving wage rates reduces mainstream costs to approximately 3,555 U.S. dollars (a 7.1 percent decline) while mediation falls to 91 U.S. dollars. Under Scenario B, doubling travel distances raises mainstream costs to approximately 4,100 U.S. dollars while mediation rises only to 112 U.S. dollars. Under Scenario C, eliminating lawyer fees entirely reduces mainstream costs to approximately 2,028 U.S. dollars while mediation remains at 103 U.S. dollars. Under Scenario D, using the statutory minimum wage of approximately 11 U.S. dollars per day (337 baht) rather than the NSO average of approximately 27.50 U.S. dollars per day (831 baht) as the shadow price reduces mainstream costs to approximately 3,460 U.S. dollars. In every scenario, mediation costs remain at least an order of magnitude below mainstream costs, and the cost reduction never falls below 94 percent.

**Table 6:** Sensitivity analysis: systemwide cost per case under alternative assumptions (U.S. dollars, 2026).

Scenario	Mainstream cost	Mediation cost	Reduction (%)
Baseline	3,828	103	97.3
A: Wages halved	3,555	91	97.4
B: Travel doubled	4,100	112	97.3
C: No lawyer fees	2,028	103	94.9
D: Minimum wage	3,460	96	97.2

Sensitivity analyses confirmed that the observed cost differentials were not artifacts of specific parameter choices. When wage rates are halved, private costs in the mainstream process decline modestly, but institutional costs remain largely unchanged because they depend on salary structures rather than the disputants’ opportunity costs. Doubling travel distances significantly increases mainstream costs but increases mediation costs only marginally because mediation involves fewer visits. Eliminating lawyer fees in a hypothetical scenario where defendants choose to self-represent reduces private mainstream costs; however, even under this assumption, the systemwide cost of mainstream processing remains an order of magnitude higher than that of mediation. When the opportunity costs of time are recalibrated using lower shadow prices for informal-sector employment, the relative savings from mediation remain above 95 percent.

These robustness checks suggest that the cost advantages of mediation were structural. They arise from the architecture of the process and not from the contingent parameters. Mediated cases require only a single coordinated interaction, whereas mainstream cases incur costs across multiple stages. This structural logic holds across all the cost assumptions tested.

### 5.6 Monte Carlo uncertainty quantification

To assess whether the cost differential is robust to sampling uncertainty in the private cost components, a Monte Carlo simulation was conducted with 10,000 replications. In each draw, private cost components (travel, food, and communication at each procedural stage) were sampled from lognormal distributions calibrated to the survey means and standard deviations reported in Table 7. Lognormal distributions were chosen because the underlying cost data are right-skewed and bounded below by zero. Government cost components, being deterministic budget-to-caseload ratios, were held fixed across all draws. Lawyer fees and opportunity costs were likewise held at their baseline values.

**Table 7:** Descriptive statistics for private cost components (baht, pre-adjustment).

Component	Min	Max	Mean	Median	SD
Police stage					
Processing time (days)	0.5	2,555	9.9	1	103.1
Contact days	0.5	50	1.6	1	2.1
Travel (baht)	4	5,000	188	100	373
Accommodation (baht)	30	4,000	869	450	1,046
Food (baht)	8	1,500	144	100	177
Telephone (baht)	2	500	61	29	81
Prosecutor stage					
Processing time (days)	0.5	730	36.9	2	110.2
Contact days	0.5	480	3.8	1	26.6
Travel (baht)	10	5,000	203	100	429
Food (baht)	17	1,000	111	100	119
Telephone (baht)	4	400	74	42	104
Court of first instance					
Processing time (days)	0.5	3,650	115.6	30	259.7
Contact days	0.5	208	3.8	1	13.0
Travel (baht)	4	10,000	247	100	565
Accommodation (baht)	2	4,000	610	250	970
Food (baht)	10	3,000	153	100	235
Telephone (baht)	5	500	58	30	74

Table reports only private cost components, which were computed as survey means and therefore exhibit sampling variation. Government cost components (police: approximately 854 U.S. dollars per case in 2026 prices, or 25,798 baht; prosecutor: approximately 364 U.S. dollars, or 10,986 baht; court: approximately 175 U.S. dollars, or 5,290 baht) are deterministic budget-to-caseload ratios with no sampling variance. All figures are in pre-adjustment baht; see Section 4 for CPI conversion.

**Figure 3:**
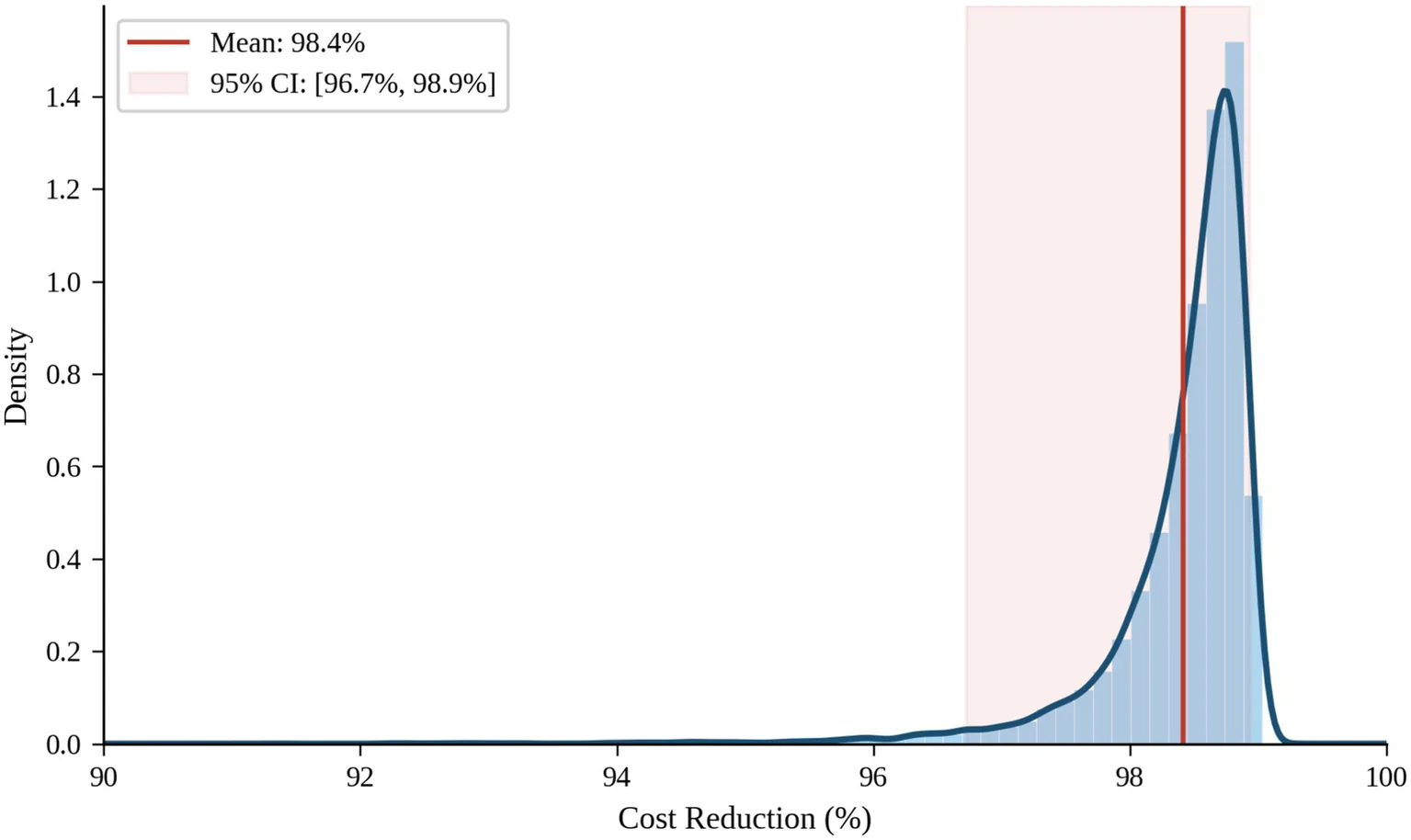
Distribution of cost reduction from Monte Carlo simulation (10,000 replications). The solid red line marks the mean reduction (98.4 percent); dashed red lines and the shaded region mark the 95 percent confidence interval [96.7, 98.9 percent].

**Figure 4:**
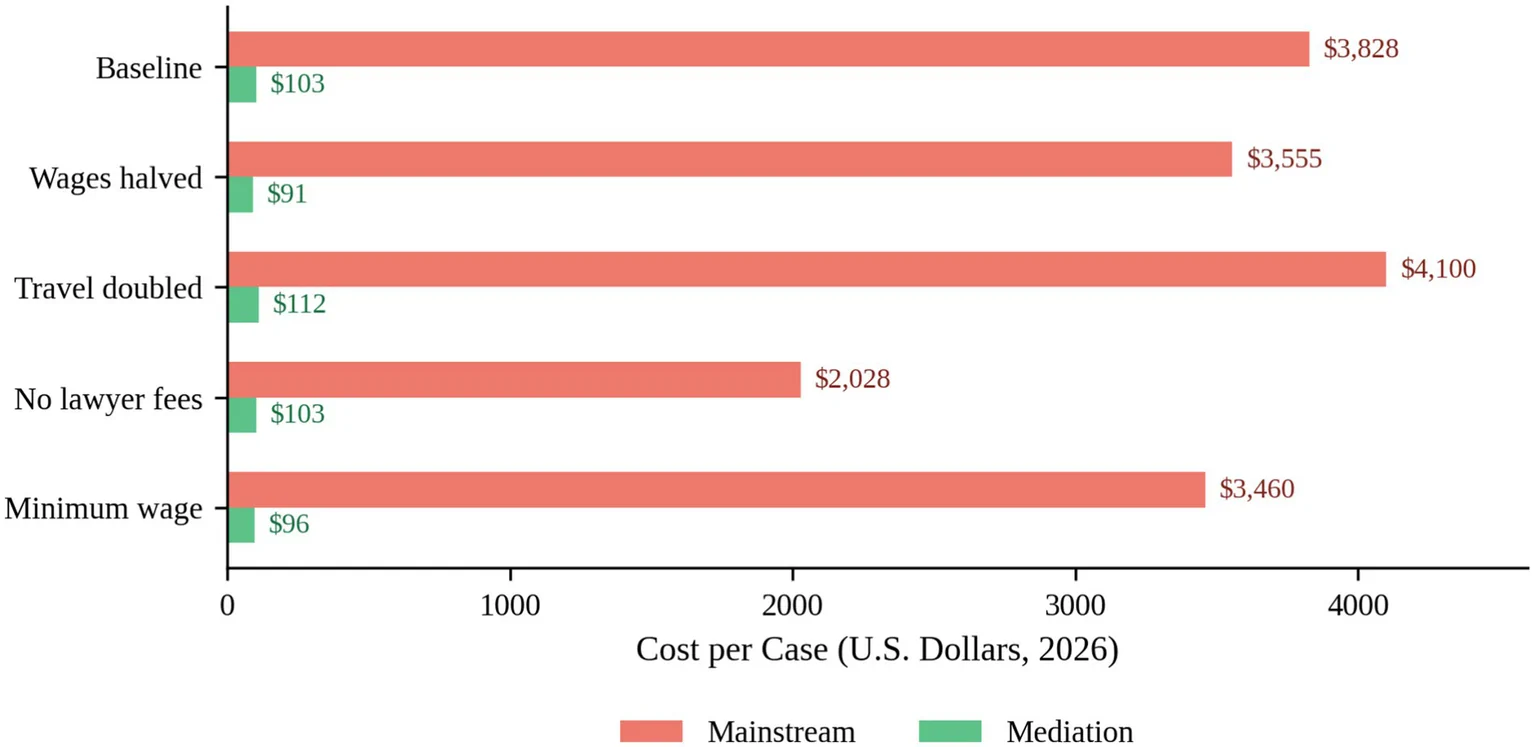
Sensitivity analysis comparing mainstream (red) and mediation (green) costs under alternative assumptions, in 2026 U.S. dollars.

**Figure 5:**
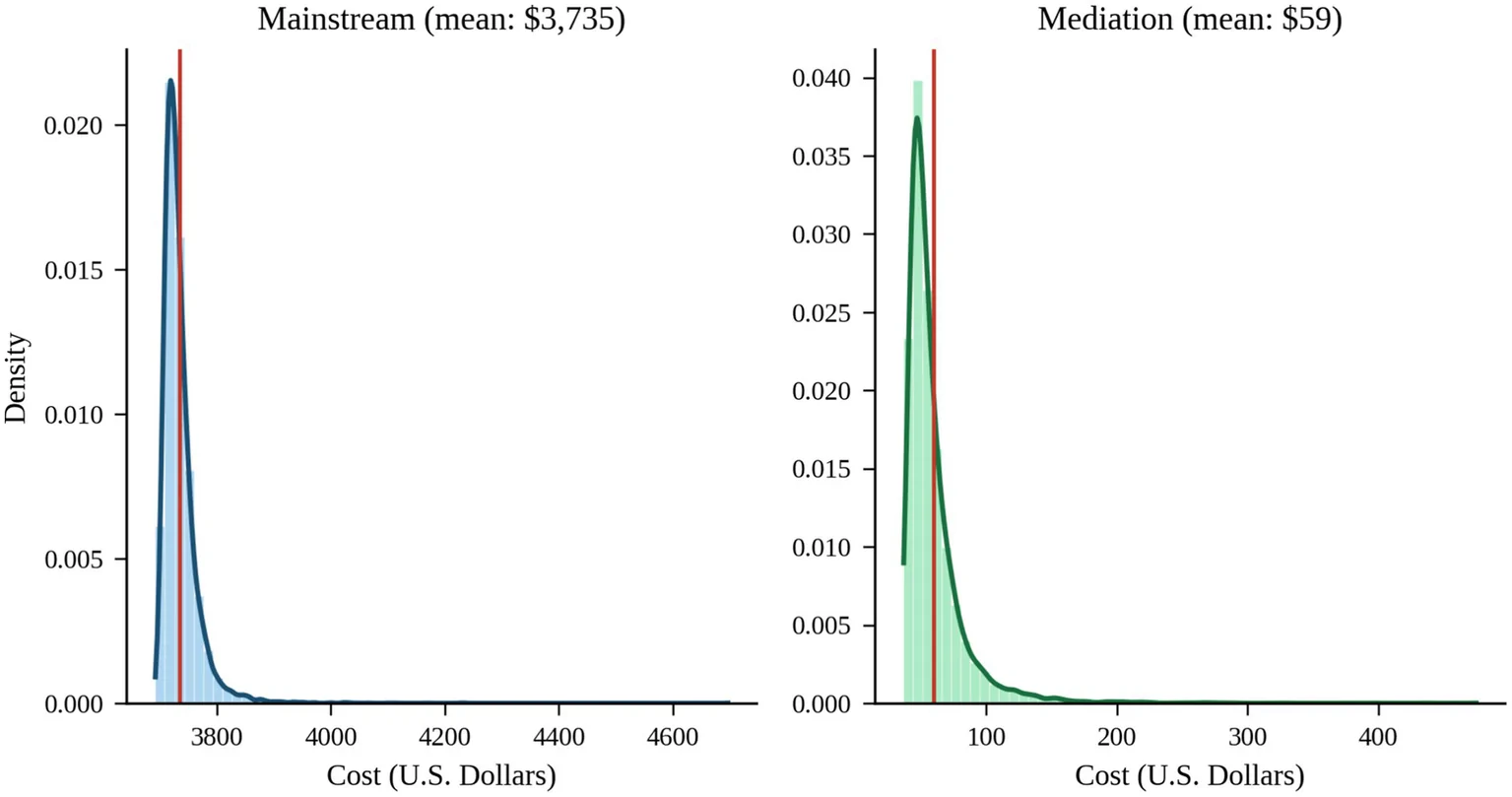
Simulated cost distributions by pathway (10,000 Monte Carlo replications). Note: Left panel is mainstream criminal justice (mean 3,735 U.S. dollars). Right panel is mediation (mean 59 U.S. dollars). Red lines mark the mean.

The simulation yields a mean mainstream cost of 3,735 U.S. dollars with a 95 percent confidence interval of [3,703, 3,811], and a mean mediation cost of 59 U.S. dollars with a 95 percent confidence interval of [40, 122]. The mean cost reduction is 98.4 percent, with a 95 percent interval of [96.7, 98.9] percent. Across all 10,000 draws, the cost reduction never falls below 87.3 percent, and the probability that the reduction exceeds 90 percent is 100.0 percent. Figures 3–5 illustrate these distributions. These results confirm that the headline finding, a cost reduction of approximately 97 percent, is not an artifact of point estimation but holds with high probability across the full range of plausible private cost realizations.

The tightness of the confidence interval reflects a structural feature of the cost comparison: government costs, which account for roughly three quarters of total mainstream costs, are deterministic and therefore contribute no sampling uncertainty. Private cost components, though individually dispersed, are small relative to the total and partially offset one another when summed across stages. The result is that even under extreme private cost draws, the mainstream total remains an order of magnitude above the mediation total.

### 5.7 Structural interpretation

A pattern emerges in the results, circling back to the basic economics established earlier. When the formal route carries heavy costs, as it plainly does here, only a fairly small set of disputes can realistically endure it; most people try to avoid a process that drains time and money long before it produces an outcome. Mediation shifts calculations. By reducing the burden on both sides, settlement is a sensible option in cases where it would otherwise be pushed further into the system. What follows from this is largely institutional. A considerable number of minor cases can be settled without disturbing the safeguards that matter, and doing so frees the police, prosecutors, and courts from concentrating on cases in which adjudication is genuinely indispensable. This reallocation of attention is less about grand reform and more about keeping everyday institutional work manageable. This is the sort of practical capacity that long-term governance frameworks, including SDG discussions, tend to worry about, even if they rarely describe it in these terms.

## 6 Discussion

### 6.1 Interpretation of findings

The pattern that emerges from the empirical results is neither subtle nor variable across the case types examined. The gap between the mainstream criminal process and mediation is large and persistent. This is not something that can be explained by a few outlier cases or a quirk in the data. This stems from the manner in which each pathway was built. The formal route requires several actors and a fair amount of administrative work at every stage, and these demands accumulate rapidly. Mediation reorganizes the sequence entirely, bringing everyone together once in a structured conversation, and ending the matter there. This difference in design explains the difference in costs more than anything else. Thus, the results must be read against the conceptual ground laid earlier rather than as a simple cost comparison.

One lesson from the economics of litigation is that the path a dispute often takes depends less on the size of the claim than on the cost of resolving it. In the formal system, the expected value of pushing a case through the police, prosecutors, and courts depends heavily on the cost terms. With rising time, travel, legal, and administrative costs, the area in which settlement is feasible begins to shrink. In Thailand’s minor criminal cases, these costs have accumulated quickly. An empirical estimate of more than 3,800 U.S. dollars per case illustrates why many disputants, especially those with limited means, find the adversarial process difficult to sustain. The system is not simply expensive; for low-stakes matters, it is misaligned with the situations that people encounter.

Mediation would reshape this dynamic under these conditions. The costs on both sides fall so sharply that the decision environments start to appear different. When private and institutional costs are compressed, the region in which settlement makes sense broadens dramatically. The cost differences documented in Section 5, approximately 97 percent across all categories, are of a scale that would shift behavior, not by changing the underlying merits of the case but by altering what is required to resolve it. This is precisely what the earlier conceptual model expected. The equilibrium would shift not because the expected judgment changes but because the cost landscape does.

There is also an access-to-justice dimension in which the cost numbers are clearer. The mainstream process that currently operates in minor criminal matters is difficult to use. The time spent traveling, hours waiting outside the courtrooms, legal fees, and income lost from missed workdays all add up. These burdens fall most heavily on the people who are least able to absorb them. This is the kind of procedural friction that Barendrecht et al. (2006) have pointed to: formal rights exist on paper, but the pathway to exercise them is steep. It is not surprising that some victims withdraw or defendants look for the quickest exit, even when that exit may be uneven or unsatisfactory.

Mediation lowers these barriers almost by design. When the mainstream pathway requires several institutional contacts, mediation requires only one contact. When formal hearings involve long waiting times, mediation sessions tend to be shorter and more predictable. This simplified structure opens the door to those who would otherwise be deterred by the cost of participation. What the cost differentials show, therefore, is not just a cheaper alternative, but a more usable one, an institutional design that restores the possibility of pursuing justice without having to pay a prohibitive price in time or money.

The sustainability argument is on a different axis. Justice institutions deal with rising caseloads, tighter budgets, and greater public scrutiny. In such environments, the ability to handle routine matters without draining capacity is not a side issue, but a condition for institutional health. Thailand’s mainstream criminal process, particularly for minor cases, requires more administrative attention than the gravity of these cases would ordinarily justify. When too many of these matters accumulate, they push against the system’s limits, slowing police work, stretching prosecutors thin, and crowding court schedules. None of these factors is conducive to the long-term performance of institutions.

Mediation offers a practical way of relieving this pressure. The model suggests that even moderate shifts toward mediation could release substantial resource savings that could be channeled into areas where they are genuinely needed, such as victim support, investigative quality, and court administration. The result is not simply cost savings in the abstract but a redistribution of institutional effort that strengthens the system’s ability to handle its core responsibilities. In this sense, mediation aligns naturally with the concerns found in the sustainable governance literature and in the broader discussions around SDG 16, although mediation’s contribution is much more operational than rhetorical.

Collectively, these findings suggest that mediation in Thailand occurs on two levels. At the interpersonal level, it offers a way to address harm and to repair damaged relationships. At the institutional level, it relieves pressure on a system that is doing too much with too few resources available for the types of cases involved. The cost differences are impressive; they show how the system might rebalance itself if the mediation is used more consistently. By widening the space in which a settlement is feasible, mediation reshapes both individual case paths and broadens the distribution of institutional work. This dual effect is what makes it significant not only for the disputants who use it but also for the long-term functioning of the justice system as a whole.

### 6.2 Limitations

None of these advantages imply that mediation is a cure-all. Restorative processes rely on voluntary and balanced participation, and can falter when these conditions are not present. Ashworth (2001, 2002) and Braithwaite (2002) cautioned against the risks of superficial settlements or power imbalances that remain unaddressed (see also Wojciechowski, 2026). The Thai system is facing similar challenges. Mediation requires trained and qualified facilitators and institutional safeguards and cannot be scaled without attention to quality. However, these limits do not diminish its value. Instead, they highlight the need for thoughtful integration and proper oversight so that the benefits identified in the empirical analysis are not offset by unintended consequences.

The administrative data used in this study do not include individual-level recidivism, compliance with mediated agreements, or participant satisfaction measures, and this is an important limitation that future research should address. Two aggregate indicators from related administrative sources, however, offer partial reassurance. First, mediation agreement rates suggest that structured dialogue produces workable outcomes in a substantial share of cases: court-annexed mediation achieves a 50.1 percent agreement rate, while community justice mediation reaches 71.6 percent. Second, absconding rates are comparable across pathways: among 2,739 individuals processed through alternative justice measures over a three-year period, the flight rate was 0.55 percent, compared with 0.43 percent in the mainstream system (data from the Rights and Liberties Protection Department and the Office of the Judiciary, respectively). These near-identical rates indicate that mediation does not compromise the system’s ability to retain participants, although they do not speak to the quality or durability of the agreements reached. A further limitation concerns data vintage. The underlying quantities were collected in 2016 and 2017, and although all monetary values are updated to 2026 prices using the consumer price index, the adjustment cannot capture structural changes in procedures, caseloads, or fee schedules that may have occurred since collection. The estimates should therefore be read as the most recent comprehensive cost accounting available rather than as a real-time measurement.

## 7 Conclusions and policy implications

What the study eventually makes clear is something that practitioners in Thailand have long suspected but have not had the chance to quantify with much precision. Once minor criminal disputes enter the formal system, they accumulate a set of burdens that were never designed with these types of cases in mind. The estimates presented earlier, the interviews, trips, repeated drafting of files, and long waits outside courtrooms, simply give measured form to what has been felt informally for years. By comparison, mediation looks almost like an entirely different institution, lighter, quicker, and far less demanding for both people and agencies. The contrast is sufficiently vast that it is no longer meaningful to describe the two as variants of the same process.

Economic theory helps explain why these patterns appear as strongly as they do. High litigation costs push people to continue or abandon cases for reasons that are often unrelated to the underlying disputes. The Thai data reflect this dynamic faithfully. If the cost of staying in the formal process routinely exceeds several thousand U.S. dollars, few find the path tolerable. When mediation lowers these costs, the decision environment shifts; settlement begins to look less like a compromise, and more like the only practical way forward. The models presented in Section 2 predict and confirm this shift.

However, the issue is not merely strategic behavior. In practice, access to justice often collapses under the weight of ordinary inconveniences, such as lost day’s wages, unexpected travel costs, and missed shifts. These sorts of costs rarely enter legal debates but determine similar outcomes. Mediation changes the cost calculus. A single session, no need for legal counsel in most cases, and minimal waiting are not marginal improvements. For many people, these factors make the difference between participating in the system and stepping away from it entirely.

The results have straightforward, albeit uncomfortable, implications for institutions. A justice system that devotes so much energy to handling low-severity matters eventually struggles with cases that require attention. Thailand is not unique in this respect; however, the strain is visible. When used sensibly, mediation eases that pressure. It frees scarce administrative time, which can be redirected toward areas where delays or oversight have real consequences. This is a form of sustainability that rarely appears in policy statements but is fundamental to how institutions remain functional year after year.

From a policy perspective, this lesson is neither radical nor complicated. Mediation structures already exist: community centers, provincial offices, and court-annexed programs, but they operate unevenly. More deliberate early referral, clearer guidance for frontline officials, and steady investment in mediator training would allow the system to use the tools it already has. The saving generated from such a shift, while significant, is not the point in itself. Its value lies in the institutional breathing room created.

However, mediation cannot be used to address every dispute. Some cases deserve fuller protection in the adversarial process, and safeguards must remain in place to maintain voluntary and fair mediation. These limitations are real, and any reform must respect them. However, recognizing limits is not the same as denying value.

Taken together, the analysis shows that mediation plays two roles simultaneously. First, it helps people resolve the types of conflicts that arise in everyday life. Second, it gives justice institutions a way to cope with volumes of minor cases they were not built to absorb. Both outcomes matter. Furthermore, both suggest that mediation should be understood not merely as an ‘alternative’ but as a practical component of a justice system trying to stay balanced under long-term pressure.

## Data Availability

The data analyzed in this study is subject to the following licenses/restrictions: the administrative microdata used in the analysis were shared with the author under a restricted arrangement with the Office of Justice Affairs. As the dataset contains non-public administrative information, it cannot be made openly available by the author. Requests for access can be directed to the Office of Justice, which is subject to internal data-governance procedures. Requests to access these datasets should be directed to saraban@oja.go.th.
